# Axillary pilonidal sinus: an unusual presentation: a case report with literature review

**DOI:** 10.1093/omcr/omae123

**Published:** 2024-10-26

**Authors:** Abdulwahid M Salih, Ronak S Ahmed, Hardi M Zahir, Yadgar A Saeed, Halkawt O Ali, Aso S Muhialdeen, Saeed H Ali, Kayhan A Najar, Fakher Abdullah, Fahmi H Kakamad

**Affiliations:** Smart Health Tower, Scientific Affairs Department, Madam Mitterrand Street, Sulaymaniyah, Kurdistan 46001, Iraq; College of Medicine, University of Sulaimani, Madam Mitterrand Street, Sulaymaniyah, Kurdistan 46001, Iraq; Smart Health Tower, Scientific Affairs Department, Madam Mitterrand Street, Sulaymaniyah, Kurdistan 46001, Iraq; Shahid Nabaz Dermatology Teaching Center for Treatment of Skin Diseases, Dermatology Department, GCWR+WC7 Sulaymaniyah, Kurdistan 46001, Iraq; Smart Health Tower, Scientific Affairs Department, Madam Mitterrand Street, Sulaymaniyah, Kurdistan 46001, Iraq; Smart Health Tower, Scientific Affairs Department, Madam Mitterrand Street, Sulaymaniyah, Kurdistan 46001, Iraq; Smart Health Tower, Scientific Affairs Department, Madam Mitterrand Street, Sulaymaniyah, Kurdistan 46001, Iraq; Smart Health Tower, Scientific Affairs Department, Madam Mitterrand Street, Sulaymaniyah, Kurdistan 46001, Iraq; Smart Health Tower, Scientific Affairs Department, Madam Mitterrand Street, Sulaymaniyah, Kurdistan 46001, Iraq; Smart Health Tower, Scientific Affairs Department, Madam Mitterrand Street, Sulaymaniyah, Kurdistan 46001, Iraq; Kscien Organization, Scientific Research Department, Hamdi Street, Azadi Mall, Sulaymaniyah, Kurdistan 46001, Iraq; Kscien Organization, Scientific Research Department, Hamdi Street, Azadi Mall, Sulaymaniyah, Kurdistan 46001, Iraq; Smart Health Tower, Scientific Affairs Department, Madam Mitterrand Street, Sulaymaniyah, Kurdistan 46001, Iraq; College of Medicine, University of Sulaimani, Madam Mitterrand Street, Sulaymaniyah, Kurdistan 46001, Iraq; Kscien Organization, Scientific Research Department, Hamdi Street, Azadi Mall, Sulaymaniyah, Kurdistan 46001, Iraq

**Keywords:** PNS, treatment, atypical location, surgical therapy

## Abstract

Despite its rarity, pilonidal sinus (PNS) in atypical locations poses significant diagnostic challenges, underscoring the need for early identification and appropriate treatment strategies. This case highlights a rare occurrence of a PNS in the axilla, emphasizing the importance of recognizing uncommon presentations of common ailments. A 27-year-old male presented with a 13-year history of painless axillary discharge, diagnosed with PNS based on clinical evaluation. Surgical excision under local anesthesia successfully treated the condition, showcasing the effectiveness of tailored management in addressing rare presentations of PNS. Surgical therapy for axillary pilonidal sinus enables complete resection and provides precise histopathological diagnoses, making it a suitable treatment option, particularly for cases involving atypical locations.

## Introduction

Pilonidal sinus (PNS) is a common perianal condition, especially in developing countries. It is formed by the ingrowth of hair, forming a foreign-body granuloma. PNS may manifest in various areas such as the hands, intermammary region, suprapubic area, umbilicus, nose, interdigital web, groin, face, neck, prepuce, penis, postauricular region, preauricular region, submental area, clitoris, scalp, and endoanal region. Surgical excision under local anesthesia is the primary treatment, emphasizing meticulous removal of the sinus. In contrast to the more typical occurrence of pilonidal disease in the sacrococcygeal area, axillary involvement represents an exceptionally rare presentation, introducing intricacies to both diagnosis and treatment of this condition [1–3].

The existing literature on its frequency, clinical attributes, and optimal management of axillary PNS is sparse, with only 12 reported cases in authentic English scientific literature [2–8].

This case was written following CaReL guidelines and includes a literature review that emphasizes the need for further research to determine the etiology, epidemiology, and standardized management approaches for axillary PNS [9]. Additionally, increased awareness of this rare presentation can aid in timely diagnosis and appropriate intervention, ensuring favorable outcomes for affected individuals.

## Case report

### Case presentation

A 27-year-old male presented with a painless axillary discharge for 13 years, however, he did not seek any medical attention before as he didn’t consider his condition serious. He would simply clean any discharge and move on, that was until two weeks ago when the discharge increased in amount prompting him to seek medical attention for the first time. The condition was associated with skin redness around the drainage site and occasional itching. The past medical and surgical histories were unremarkable. The family history was insignificant as well.

### Clinical examination

A painless swelling with a central hole was detected at the posterior aspect of the axilla. In addition, no discharge or lymphadenopathy was noted.

### Diagnostic approach

Based on clinical evaluation, including feeling the cyst and tract, and drainage, the patient was diagnosed with PNS of the axilla, and no further investigations were deemed necessary.

### Therapeutic interventions

**Figure 1:**
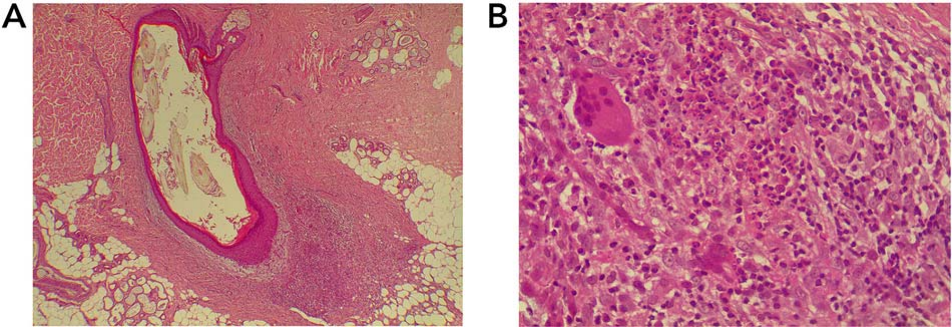
(**A**) The section shows a cavity lined by keratinizing stratified squamous epithelium containing in its lumen multiple hair shaft fragments. There is a surrounding heavy mixed inflammatory infiltrate with fibrosis. (**B**) The inflammatory infiltrate includes neutrophils, lymphocytes, plasma cells, and multinucleated giant cells, along with capillary proliferation lined by activated endothelial cells. [Hematoxylin and eosin; original magnification ×40 (**A**) and ×400 (**B**)].

In the supine position under local anesthesia, the sinus, and the entire tract were excised to remove the hair and a hair punch from the sinus. The wound was treated with Salih’s mixture, which is prepared using 100 g petroleum jelly, 50 g henna powder (*Lawsonia inermis* powder), and 5 g tetracycline. The wound was then covered with Kurdish gum, a natural resin obtained from the mastic tree (*Pistacia lentiscus*) [10]. Histopathologic examination of the specimen showed a cavity lined by keratinizing stratified squamous epithelium containing in its lumen multiple hair shaft fragments with a surrounding heavy mixed inflammatory infiltrate with fibrosis (Fig. 1).

### Follow-up

**Figure 2:**
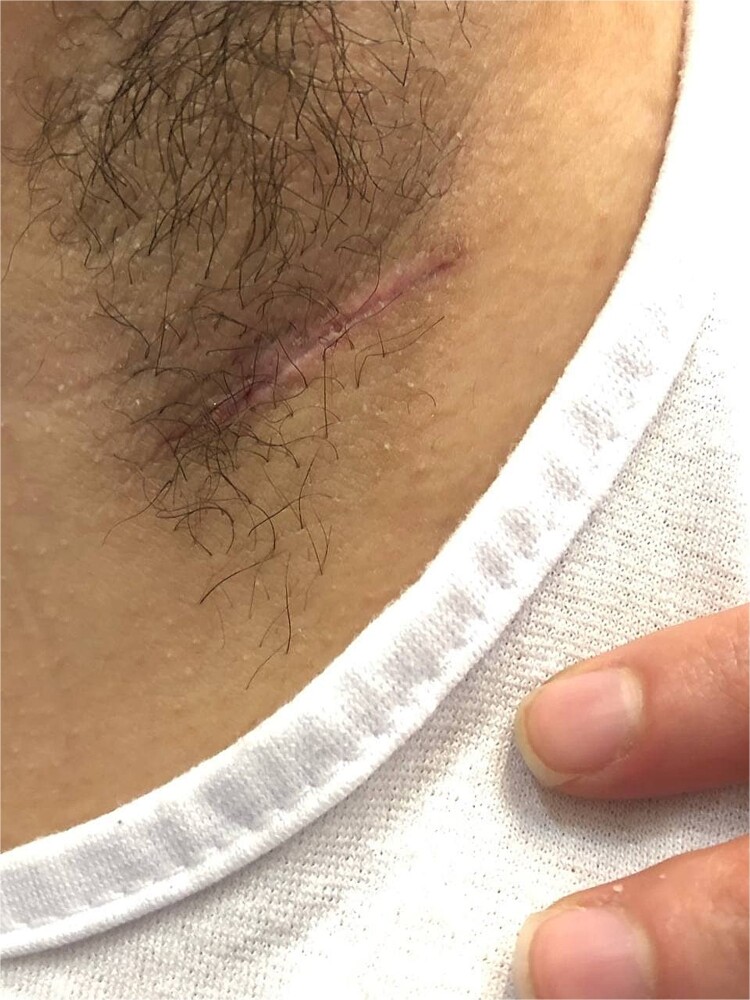
The surgical incision site of the axillary pilonidal sinus.

The postoperative period was uneventful. One month later, the wound healed completely with no significant residual pathology (Fig. 2).

## Discussion

Pilonidal sinus is rarely encountered in the axilla, which was first described in the 1950s by Arid et al.; however, since that time, only 12 cases have been documented in the scientific literature, summarized in (Table 1). The first case reported by Arid et al. was of a 34-year-old man with symptoms of edema and purulent discharge from a sinus in the right axilla. Numerous attempts to excise the inflamed region proved unsuccessful. Ultimately, a radical excision was undertaken, involving the complete removal of the skin from the axillary floor, including all underlying connective tissue and the entire area affected by axillary suppuration. Histopathological examination was confirmatory of PNS with the presence of foreign body reaction and hair in the specimen, further supporting the diagnosis [3]. In the study by Chen et al., a 21-year-old female complained of a 5-year history of a pit and nodule in the left axilla, but it later progressed to suppurative discharge, and the patient sought medical help where surgery was advised with primary wound closure, and histopathology showed granulation tissue [7]. In accordance with Chen et al.’s patient, the present patient complained of symptoms of discharge, redness, and itching for multiple years and sought medical attention later during the disease course. During the literature review, most of the patients complained of symptoms for multiple months or even years, and then they were diagnosed with axillary PNS. Contrary to the other cases in the literature, the present case, after surgical excision of the sinus that contained hair as well, the wound was treated with Salih’s mixture and covered with Kurdish gum. This technique has been used in PNS in various other locations with good outcomes [10].

**Table 1:** Review table

**Author/year of publication**	**Country**	**Type of Study**	**No. of cases**	**Age (years)**	**Sex**	**Presenting symptom**	**Side**	**Management**	**Macroscopic hair finding**	**Histopathology**
Arid et al./1952 [3]	UK	*	1	34	M	Free discharge of pus from the sinus and some edema around it.	R	Multiple unsuccessful attempts at excision of the inflamed area were undertaken; finally, a radical excision of the skin of the floor of the axilla with all connective tissue underlying it and with the whole area of axillary suppuration was done.	-	The cavity was of irregular shape and was packed with polymorphs and macrophages. Two hairs were found in the track, both surrounded by a foreign-body reaction. The appearances are those of a pilonidal sinus.
Ohtsuka et al./1993 [4]	Japan	*	2	21	F	Intermittent pain and purulent discharge for three months.	L	Fistulectomy and opening of the two sinuses.	Four hairs in the sinus.	Several hair follicles were present in the depths of the sinus.
				22	F	Intermittent, severe pain and discharge for three months.	R	Surgery was done through Two incisions parallel to the axillary wrinkle lines were performed to prevent a postoperative hypertrophic scar.	One hair with a barely bulb-like end at its proximal end was seen in the track.	The proximal end of the buried hair seemed to consist of a half-destroyed hair follicle.
Ohtsuka et al./1994 [5]	Japan	*	5	21	F	Mildly painful induration in axilla for 6 months.	R	Fistulectomy through Two incisions parallel to the axillary wrinkle lines.	One hair was identified in the distal sinus.	Two hairs in the sinus.
				21	F	Intermittent pain and purulent discharge.	R	Fistulectomy with T-shaped incision including 2 openings of sinuses.	Two hairs in the proximal sinus.	One hair in the sinus with irregular granulation tissue and foreign body reaction.
				17	F	Purulent discharge either spontaneously or under finger pressure.	R	Surgical management but unspecified.	Hair was detected but unspecified number.	No hair was detected, mild to moderate inflammatory or foreign body reaction.
				21	F	-	L			Hair was detected, mild to moderate inflammatory or foreign body reaction.
				30	F	-	R			No hair was detected, mild to moderate inflammatory or foreign body reaction.
Sengul et al./2009 [2]	Turkey	*	1	25	F	Intermittent small amount of leakage during the past year	R	Total excision of the tracts with the neighboring subcutaneous tissue and the elliptical skin part containing the sinus orifice.	No hair was detected.	Hair in the sinus tract.
Sion-Vardy et al./2009 [6]	Israel	Retrospective case review	2	19	M	-	-	Surgical management.	Hair was detected.	Confirmed but not specific finding provided.
				22	F	-	-	Surgical management.	Hair was detected.	Confirmed but not specific finding provided.
Chen et al./2020 [7]	China	*	1	21	F	Asymptomatic pit and subcutaneous nodule for 5 years, then became painful, swollen, and suppurated during the last 2 years, followed by spontaneous ulceration and drainage.	L	Scarring tissue and sinus tract were completely excised after injecting the tract with methylene blue, followed by primary wound closure.	Hair not detected.	A sinus tract surrounded by granulation tissue and squamous epithelium with mild chronic inflammatory infiltrate and a few hair follicles in the subcutaneous tissue.

Abbreviations: R = Right, L = Left, M = Male, F = Female, * = case report, UK = United kingdom.

The differential diagnosis of axillary PNS is broad; for instance, Arid et al. conducted tuberculosis testing on the purulent discharge from the axillary sinus and the pleural fluid for tuberculosis. However, the results came back negative. Other microbes like actinomyces can potentially cause such a presentation as well. Axillary PNS can be misdiagnosed as hidradenitis suppurativa, folliculitis, and ruptured epidermal inclusion cyst. Despite these conditions being more common, axillary PNS should still be recognized by clinicians as a possible cause in such scenarios [2, 3].

In 10 cases, the laterality of the axillary PNS was noted, of which 70% were located on the right side. Although more data is needed to further support this observation, perhaps this might provide some clue that more friction and usage of the dominant right arm can be a part of the pathophysiology of PNS formation in the axilla.

Surgical intervention, mirroring the approach for sacrococcygeal PNS, stands as the cornerstone of axillary PNS management. However, anatomical variations and the presence of surrounding structures necessitate adaptations in the surgical strategy. Local anesthesia plays a crucial role in this meticulous process, allowing for precise identification and complete removal of the sinus tracts and any associated hair follicles, ultimately aiming for a successful outcome [3–7].

## Conclusion

In summary, the case underscores the importance of identifying and managing rare occurrences of pilonidal sinus, especially in unconventional locations like the axilla. Diagnostic challenges highlight the need for thorough evaluation to avoid treatment delays. Surgical intervention remains pivotal, with adaptations needed for anatomical variations. Further research is warranted to optimize treatment strategies and improve patient outcomes.
